# Efficacy and Safety of Nasal Immunisation with Somatostatin DNA Vaccine for Growth Promotion in Fattening Pigs

**DOI:** 10.3390/ani12223072

**Published:** 2022-11-08

**Authors:** Chao Chen, Zichao Zhou, Kaifeng Niu, Chao Du, Aixin Liang, Liguo Yang

**Affiliations:** 1 National Center for International Research on Animal Genetics, Breeding and Reproduction (NCIRAGBR), College of Animal Science and Technology, Huazhong Agricultural University, Wuhan 430070, China; 2 Hubei Province’s Engineering Research Center in Buffalo Breeding and Products, Wuhan 430070, China

**Keywords:** DNA vaccine, fattening pig, biosecurity, somatostatin, growth

## Abstract

**Simple Summary:**

Growth qualities are critical economic traits that substantially impact pork yield. Here, we evaluated the effect of somatostatin (SS) DNA vaccines on growth promotion in fattening pigs. Our results demonstrate that immunisation with SS DNA vaccine induces cellular and humoral immunity in fattening pigs, producing anti-SS antibodies that neutralise endogenous SS, thereby promoting daily gain and slaughter weight. In addition, the vaccine does not pose a safety challenge. Consequently, it is possible to accelerate the growth of fattening pigs through the immune SS DNA vaccine, which will bring more economic benefits to the company and the farmer.

**Abstract:**

This study aimed to evaluate the efficacy and safety of the SS DNA vaccine on growing pigs. Randomly, 147 pigs were divided into four groups, treatment 1 (T1, 3 × 10^9^ CFU/mL, *n* = 39), T2 (3 × 10^8^ CFU/mL, *n* = 35), T3 (3 × 10^7^ CFU/mL, *n* = 35) and control group (phosphate-buffered saline, *n* = 38). All animals received two vaccinations separated by 45 days and the same diet and management. The results showed that all treatment groups (T1, T2 and T3) had significantly higher slaughter weight (d 185) than the Ctrl group (*p* < 0.05), and daily gain between 50 and 110 days of age was significantly higher in all treatment groups than in the Ctrl group (*p* < 0.05). Antibody-positive pigs have significantly higher daily weight gain than that in antibody-negative pigs (*p* < 0.05). The results of the meat quality analysis showed no significant changes between the P (antibody-positive pigs) and N (antibody-negative pigs) groups. Furthermore, the results showed that antibody titres at 110 and 185 days had a significant positive correlation with the daily weight gain (*p* < 0.05) and a significant negative correlation with the backfat thickness (*p* < 0.05). Evaluating the safety of vaccines by PCR amplification of target genes (GS/2SS), faecal, soil and water samples had no target genes detected by PCR amplification in these samples after 5 days, and no GS/2SS were detected in the blood and tissues for the experimental period. Moreover, no abnormalities were found in pathological sections of the P group compared with the N group. In conclusion, SS DNA vaccines can promote the growth of fattening pigs to a certain extent without altering the meat quality, and it has no effects on the safety of the surrounding environment.

## 1. Introduction

Growth hormone (*GH*) is a critical hormone in growth regulation, by modulating the growth hormone insulin-like growth factor (*GH-IGF)* axis and promoting cellular protein synthesis, cell division, and proliferation. Hence, it can accelerate the growth of muscle, bone, and other animal tissues [1]. Studies have shown that somatostatin (SS) has a wide range of inhibitory effects on *GH*, prolactin (*PRL*), thyroid-stimulating hormone (*TSH*), glucagon and insulin [2]. In addition, SS also inhibits digestive system functions, including digestive juice secretion, intestinal motility and blood flow in the small intestine [3,4,5]. SS exerts its biological effects mainly by binding to somatostatin receptors (*SSTRs*), which are widely expressed in the body’s tissues [6]. Passive [7] or active [8,9] immunisation against somatostatin through immunological methods using specific antibodies to neutralise endogenous somatostatin to block somatostatin-somatostatin receptor binding and promote the release of growth hormones and other hormones to improve the efficiency of nutrient uptake in animals to promote growth. The current study evaluated the effect of the SS DNA vaccine (pVGS/2SS-asd) on growth traits in fattening pigs through nasal immunisation.

It is well known that somatostatin has an inhibitory effect on animal growth. A previous study demonstrated that the production of antibodies to SS by the body exposed to active immunisation releases their growth inhibitory effect [10] and promote the growth of animals such as sheep [11,12] and heifer [8]. All of the above evidence suggests that SS-active immunisation is an effective method to promote animal growth. However, due to their high preparation costs, the promotion of synthetic peptides and recombinant protein and their application in livestock production is limited.

The advent of genetically engineered vaccines has brought new ideas to active immunisation with somatostatin. DNA vaccines have many advantages compared to conventional vaccines, including straightforward design, low production costs, ease of transport, no unsafe infectious agents involved, and encoding of multiple immunogenic epitopes [13,14]. However, DNA vaccines also have some disadvantages, such as having relatively poor immunogenicity [15], and the insertion of foreign DNA into the host genome may cause the cell to become cancerous [16]. In addition, the live attenuated Salmonella strain has the potential to spread across the natural environment through faeces; this could lead to contamination of the environment and a virulent reversion through the process of survival ex vivo [17,18]. Therefore, the objective of this study was to (1) evaluate the effects of the bacteria-delivered SS DNA vaccine on the growth of fattening pigs and (2) to evaluate the safety of SS DNA vaccine, including the surrounding environment and insertion of foreign DNA into the host genome.

## 2. Materials and Methods

### 2.1. Preparation of Bacterial Vaccine

The SS DNA vaccine (pGS/2SS-asd) was constructed previously in our laboratory using the pVAX-asd eukaryotic expression plasmid [19]. Before immunisation, bacteria were plated on LB agar in triplicate to determine the number of colony-forming units (CFU) and were adjusted approximately 3 × 10^9^ CFU/mL by PBS.

### 2.2. Animals and Immunisation Protocol

A total of 147 cross-bred starter pigs (Duroc-Landrace-Yorkshire), around 50 days old, were selected from a pig farm in Hubei, China (Hubei Jinlin Original Breed Livestock Co., Ltd.). All the animals were in good general health, and their physical condition was nearly the same size in each group. During the test period, the animals were fed and watered *ad libitum*. The barns were equipped with ventilation, pens were cleaned twice daily and animal behavioral observations were made daily. The animal handling procedures and all experimental protocols were approved by the Huazhong Agricultural University Animal Care and Use Committee (HZAUSW-2018-029).

**Figure 1:**
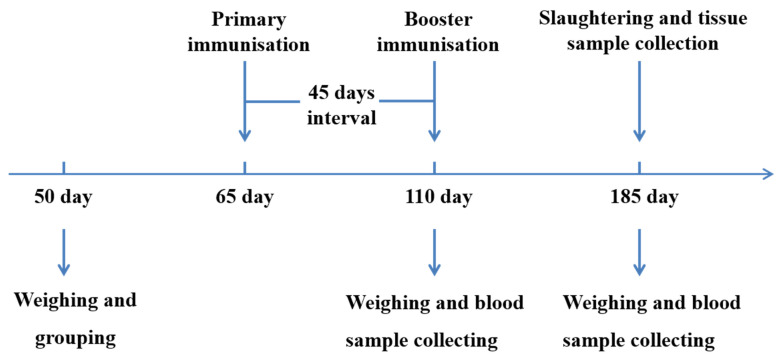
An outline explains the experimental design. Experimental pigs were immunised with SS DNA vaccine on days 65 and 110, and the control group was administered PBS. All pigs were slaughtered on days 185.

All animals were divided into four groups: groups T1 (3 × 10^9^ CFU/mL, *n* = 39), T2 (3 × 10^8^ CFU/mL, *n* = 35) and T3 (3 × 10^7^ CFU/mL, *n* = 35) were nasally immunised twice (09:00 and 15:00) a day with 15 mL of the SS DNA vaccine for 3 days, respectively. The control group (*n* = 38) was nasally immunised twice daily with 15 mL of PBS for 3 days. All animals were immunised twice with an interval of 45 days (Figure 1).

### 2.3. Blood Collection and Antibody Titre Detection

The blood samples of pigs were collected from the jugular vein on days 110 and 185 of the experiment. The serum was separated by centrifugation and stored at −80 °C until further analysis.

Indirect ELISA was used to detect SS antibody titres in pig plasma. For detailed steps refer to the previous study [20]; those that differ from them include (1) each well of the 96-well micro titre plate was coated 100 ng/100 μL of SS antigen (Sangon Biotech (Shanghai) Co., Ltd.; Shanghai, China); (2) serum samples (100 μL) diluted with PBS (1:25, 1:50, 1:100, 1:200, 1:400, 1:800, 1:1600, 1:3200, 1:6400). Finally, under the same dilution factor, if the OD 450 value of the experimental group was greater than the mean of OD 450 value in the control group + 2SD (standard deviation), then it was judged as positive [21,22].

### 2.4. Detections of Hormones

ELISA Kit (MLBIO Biotechnology Co., Ltd.; Shanghai, China) specific to porcine was used to measure *IL-4* (mL002314), *IFN-γ* (mL002333), *GH* (mL002349) and *IGF-1* (mL002344) concentrations. For specific operation methods, refer to the product manual. The intra- and inter-assay coefficients of variation were less than 10.0% and 10.0% for *IL-4*, *IFN-γ*, *GH*, and *IGF-1*. The assay sensitivity were 0.75 ng/mL for GH, 5 ng/mL for IGF-1 and 4 ng/mL for IL-4, 2.5 ng/mL for IFN-γ.

### 2.5. Meat Quality and Backfat Thickness Analysis

The experimental pigs were slaughtered on day 185, and the longissimus dorsi was collected and stored on ice for meat quality analysis. Backfat thickness was tested with ultrasonography before slaughter. Subsequently, ten pigs were randomly selected from each antibody-positive and -negative groups for meat quality determination. Each indicator was measured as follows: The pH value was measured at the center of the longissimus dorsi after 1 h of slaughter (pH_1_ value) and again after 24 h of refrigeration at 4 °C (pH_24_ value), and the meat was taken for pH measurement on three different sides using a pH meter; meat samples from the longissimus dorsi cross-section within 2 h of slaughter were visually inspected and scored on a 5-point scale for colour and marbling using the National Pork Producers Council’s (NPPC) Meat Colour Scale and Marbling Scale; The sample is cut into 1 cm^3^ squares and the cut resistance is measured along the vertical direction of the muscle fibres using a mass spectrometer; moisture, ash, crude protein and intramuscular fat were determined according to the method of the national standard GB5009.3-2016 “National Standard for Food Safety Determination of Moisture in Food”.

### 2.6. Environmental Safety Testing

Fusion gene GS/2SS in bacteria from faecal, water, and soil samples was detected by a PCR method to evaluate the safety of the SS DNA vaccine for the surrounding environment. The faecal, water and soil samples were collected from immunised pigs on days 3, 5, 7, 10, 15 and 30 after immunisation. Faecal (1 g) and soil aliquots (1 g) were placed into 9 mL of sterile PBS, respectively. These samples were serially diluted in a 10-fold series with sterile PBS, up to a dilution of 10^3^-fold. The 10-fold dilution series (200 µL of each dilution) were plated onto MacConkey agar (Tianhe, Hangzhou, China) and incubated at 37 °C for 24 h. Water samples (200 µL) were also plated onto MacConkey agar for incubation. All suspect colonies were picked and the target fragment GS/2SS of the vaccine plasmid DNA was detected by PCR (Primer: F: GCTGTGATGAATGAAACCGTAGA; R: GCAACAGGAGGGATACATAGAGG). Amplification was carried out under the following conditions: Pre-denaturing at 94 °C for 5 min, 35 cycles of denaturation at 94 °C for 40 s, annealing at 58 °C for 60 s, and extension at 72 °C for 40 s in a total volume of 20 µL of mixed liquids, including DNA (1 µL), RNase-free water (8 µL), sense and antisense (1 µL) and TaqMix (10 µL).

### 2.7. Data Statistics

The data were analysed using the MIXED procedure of SAS 9.4 software (SAS Institute, Inc., Cary, NC, USA). The mean values were compared using Tukey’ multiple range test with a significance level of *p* < 0.05. Nonparametric data were analysed using the chi-squared test. A bivariate analysis of Pearson correlation was performed. Variability in the data was expressed as the standard error of means (SEM), and *p* < 0.05 was considered statistically significant.

## 3. Results

### 3.1. Effect on Serum Anti-SS Antibody Titre Levels and Positivity Rate in Pigs

As shown in Table 1, anti-SS antibody titre levels in serum were analysed. On day 45 after the primary immunisation (d 110), there were no significant differences between the treatment groups (*p* > 0.05), where the highest antibody titre was in the T1 group at 268.75. In addition, there was no significant difference in antibody positivity between the groups (*p* > 0.05). On day 75 after the booster immunisation (d 185), there was no significant difference in antibody titre levels between the treatment groups (*p* > 0.05). Both antibody titres and antibody positivity rates were at low levels compared to d 110.

### 3.2. Effect of Hormone Levels in the Serum

*IL-4* and *IFN-γ* were detected by the ELISA kit (Table 2). There was no significant difference in *IL-4* levels between the antibody-positive (P) and –negative (N) groups on either day 110 or day 185 (*p* > 0.05). In addition, there was no significant difference in *IFN-γ* levels between the P and N groups at day 110 and day 185 (*p* > 0.05).

Furthermore, we measured and analysed serum concentrations of *GH* and *IGF-1* (Table 3). The results showed no significant difference in *GH* and *IGF-1* levels between the P and N groups at 110 days (*p* > 0.05). Similarly, there was no significant difference in *GH* and *IGF-1* concentrations between the P and N groups on days 185 (*p* > 0.05).

### 3.3. Effect on the Growth of the Fattening Pigs

As shown in Table 4, this study evaluated the effect of different concentrations of SS DNA vaccines on body weight and daily gain. The results revealed that the weight in pigs from the T3 group (59.44 ± 0.688) was significantly higher than the Ctrl group (56.42 ± 0.681; *p* < 0.05) at 110 days. At slaughter (d 185) in fattening pigs, weight was significantly higher in all treatment groups (T1, T2 and T3) than in the Ctrl group (*p* < 0.05). Furthermore, we analysed the daily gain of pigs at different periods. At 50 to 110 days, daily gain was significantly higher in treatment groups (T1, T2 and T3) than that in the Ctrl group (*p* < 0.05). Between 110 and 185 days of age, a significant increase in daily weight gain in the T1 and T3 groups compared to the Ctrl group (*p* < 0.05). Throughout the experimental period (days 50 to 185), the daily weight gain was significantly higher in the T1 group than that in the Ctrl group (0.74 ± 0.012 vs. 0.69 ± 0.012, *p* < 0.05).

In addition, our study analysed the daily gain of antibody-positive (P) and antibody-negative (N) pigs (Table 5). The results showed that between 50 and 110 days of age, the daily gain of the P group was significantly higher than that of the N group by 0.09 kg/d (*p* < 0.05). Compared to the N group, the daily weight gain was significantly higher in the P group between 110 and 185 days of age (*p* < 0.05). Meanwhile, the daily gain of the P group (0.75 ± 0.012) was significantly higher than that of the N group (0.71 ± 0.007) throughout the experimental period (days 50 to 185) (*p* < 0.05).

### 3.4. Effect on Meat Quality and Backfat Thickness of Fattening Pigs

Firstly, we tested the PH at 1 h and 24 h and there was no significant difference between the antibody-positive (P) and -negative (N) pigs (*p* > 0.05). No significant difference in meat colour and marbling was found between groups P and N (*p* > 0.05). Similarly, the differences in cut resistance, moisture, ash and crude protein between the P and N groups were insignificant (*p* > 0.05). Furthermore, there was no significance in intramuscular fat in the P group compared to the N group (*p* > 0.05). In addition, ultrasound was used to test the backfat thickness of fattening pigs, and the results showed that the P group was lower than that of the N group, but the difference was not significant (*p* > 0.05) (Table 6).

### 3.5. Correlation Coefficients of Antibody Titres with Daily Weight Gain and Backfat Thickness

As shown in Table 7, the correlation coefficients between antibody titres with daily weight gain and backfat thickness were analysed. Antibody titres at 110 days were significantly and positively correlated (*r* = 0.664) with daily weight gain between 50 and 110 days in fattening pigs (*p* < 0.01). Antibody titres at 185 days were significantly and positively correlated (*r* = 0.447) with daily weight gain between 110 and 185 days in fattening pigs (*p* < 0.05). Throughout the trial (d 50 to d 185), both days 110 (*r* = 0.396) and 185 (*r* = 0.446) antibody titres were significantly and positively correlated with daily weight gain (*p* < 0.05). In addition, our study results showed a significant negative correlation (*r* = −0.406) between backfat thickness and antibody titres at 110 days (*p* < 0.05).

### 3.6. Safety of SS DNA Vaccines

**Figure 2:**
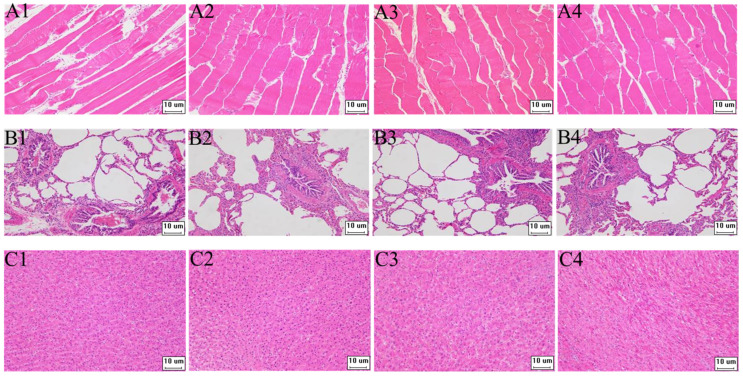
The pathological paraffin tissues section of pigs (200×). (**A**–**C**) are paraffin tissue sections of muscle, lung, and liver tissue, respectively; 1–3 are antibody-positive pigs, and 4 is antibody-negative pigs.

The target fragment GS/2SS of the vaccine plasmid DNA was amplified by PCR and no gene integration was detected within the sensitivity range of the PCR compared to the positive control (Supplementary Figure S1). Furthermore, our study investigated the safety of the environment around the barn. Soil samples were collected at 3, 5, 7, 10, 15 and 30 days after immunisation at 1, 3 and 5 m east, west, south and north of the barn and at 7 m from the air vent, respectively. The target fragment GS/2SS was not detected within the sensitivity range of PCR (Supplementary Figure S2). On the 3rd, 5th, 7th, 10th, 15th and 30th days after immunisation, faecal and water samples were collected by geometric sampling in the test pens, and the target fragment GS/2SS of the vaccine plasmid was detected by PCR. Within the sensitivity range of the PCR, the recombinant fragments were amplified on days 3 and 5 in the water samples and on day 5 in the faecal samples. However, no amplified bands were found in either the water or faecal samples after day 5 (Supplementary Figure S3). Compared with the antibody-negative pigs, the tissue morphology of all samples in the antibody-positive pigs was normal, and no pathological changes were observed (Figure 2).

## 4. Discussion

Compared to standard protein vaccinations, the clinical benefits of DNA vaccines include low cost, vaccine durability, high productivity and facile antigen customisation. On the other hand, clinical investigations revealed that the immunogenicity of DNA vaccines was relatively low [23]. In the present study, when we constructed a vaccine, the hepatitis B surface antigen gene (HBsAg-S) was inserted into the vector to address this problem. HBsAg-S can further improve immune response to DNA vaccines [24]. HBsAg-S was inserted into a gene vaccine previously constructed in the laboratory, and antibody positivity rates in serum after 14 days of immunisation ranged between 40% and 100% [25]. However, our results show antibody positivity rates between 30.77% and 40.00%. The relatively low antibody positivity rate after this study might be due to too long between immunisation and blood collection, which means our experimental design is imperfect. Wang et al. reported that antibody positivity rates were between 44.44% and 83.33% two weeks after immunisation, but between 33.33% and 61.11% four weeks after immunisation [22], suggesting a gradual decrease in antibody positivity over time. On the other hand, the dose of vaccine may also be a significant contributor to this phenomenon. Han et al. reported that oral administration of a 5 × 10^10^ CFU/mL concentration of SS DNA vaccine induced 100% antibody positivity in piglets, while 5 × 10^9^ CFU/mL and 5 × 10^8^ CFU/mL concentrations induced 30% and 20% antibody positivity, respectively [20]. Furthermore, we examined the levels of cytokines in the serum, and the results showed that the concentrations of IL-4 and IFN-γ in the P group were not significantly different from those in the N group. On the one hand, this may be because of the long interval between immunisation and blood collection. On the other hand, it may be related to the physiological hormonal compensation of the animal organism. T helper (Th) cells play a key role in regulating immune response, including th1 and th2 cells. IFN-γ production by Th1 cells is associated with cell-mediated immune function, and IL-4 produced by Th2 cells assists in the humoral immune response [26,27]. Moreover, research reported that IFN-γ and IL-4 significantly enhanced specific Th1 and Th2 cell immune responses, respectively [28]. In our study, although the insertion of HBsAg-S improved the immunogenicity of the SS DNA vaccine, a high antibody positivity rate was still not obtained. In addition to increasing the dose of the vaccine, the addition of immuno-adjuvant [29,30] is one of the important approaches to improve the antibody-positivity rate of the SS DNA vaccine.

Immunisation against SS has been reported to enhance growth in various species, including mice [31], lambs [9], rats [22], and cattle [8]. Previous studies in the laboratory have reported that the SS DNA vaccine is attributable to improving the growth performance of piglets through an influence on GH secretion [20]. In this study, we evaluated the effect of the SS DNA vaccine on growth promotion in fattening pigs. We showed that the weight at slaughter and daily weight gain throughout the feeding period was significantly higher in the SS DNA vaccine immunised group than in the Ctrl group. Several studies have shown significant positive correlations between anti-SS antibodies and average daily gain [20,32], which is consistent with our results, suggesting that the specific antibodies produced by vaccination neutralised endogenous SS in pigs and promoted growth in fattening pigs. Furthermore, the results did not reveal a significant dose-dependence of daily weight gain in fattening pigs, which is the same as the previous results of Liang et al. [33]. Because of the decrease in antibody positivity at higher doses, the loss of dose-dependence may be related to the immune stress induced by higher doses of vaccine [34]. This study is the first to analyse the meat quality after SS immunisation. The results showed no significant changes in the P group compared to the N group, including pH_1_, pH_24_, meat colour, marbling, cut resistance, moisture, ash, intramuscular fat and crude protein. Immunisation promotes the secretion of GH, which causes a decrease in intramuscular fat by stimulating lipolysis and regulating lipid deposition in adipose tissue [35], which may be an indirect cause of the significant negative correlation between backfat thickness and antibody titres.

DNA vaccine safety concerns are genetic, immunologic, toxic, and environmental [13]. Regulatory agencies, such as the European Medicines Agency (EMA), the World Health Organisation (WHO) and the Food and Drug Administration (FDA), have published guidelines for DNA vaccines [36]. Before any DNA vaccine can be submitted for regulatory approval, it needs to be tested for safety and performance [37]. In this study, genomic DNA was collected from pig blood, lung liver, and muscle tissues. No fusion gene fragment GS/2SS was detected, which is consistent with the experimental results of Liang et al. [33] and Han et al. [20]. Gene integration in DNA vaccines has not been reported to date and is theoretically less likely than the natural mutation rate of the genome [33]. However, the GS/2SS gene was observed in day 3 and 5 water samples and day 5 faecal samples. Since the study was conducted by nasal immunisation through aerosolising the bacteriological solution, it was difficult to avoid spreading in pen during the vaccination. Some studies have reported that the live attenuated Salmonella strain can spread in the natural environment by faecal [17]. Importantly, No GS/2SS gene was detected between 5 and 30 days after immunisation, suggesting that the vaccine bacterium has a limited ability to survive in the natural environment and is not able to spread over a large, prolonged period of time. Finally, compared to antibody-negative pigs, no significant pathological toxicity changes were observed in pathological sections of lung, liver, and muscle tissues in antibody-positive pigs, indicating that the SS DNA vaccine does not produce toxicity while promoting growth in fattening pigs.

## 5. Conclusions

In conclusion, nasal immunisation with SS DNA vaccine in our study resulted in the production of anti-SS antibodies in a proportion of fattening pigs, which can increase their daily gain and slaughter weight to a limited extent. Moreover, while promoting pig growth, there was no significant effect on meat quality. Finally, the vaccine safety was assessed, and no genomic integration, histopathology or contamination of the surrounding environment of animals was found. However, it needs to confirm whether optimising the sampling time between immunisations could improve the antibody positivity rate or whether further research is warranted to make this vaccine more efficient by trying vaccines with different immuno-adjuvants and routes of administration.

## Figures and Tables

**Table 1:** The antibody titre and antibody-positive rate in pigs at different doses of the SS DNA vaccine.

Groups	Age of Days			
	Day 110		Day 185	
	Antibody Titre	Positive Rate	Antibody Titre	Positive Rate
T1	268.75 ± 68.96	30.77% (12/39)	50.00 ± 21.65	20.51% (8/39)
T2	226.92 ± 47.83	37.14% (13/35)	55.00 ± 18.37	14.29% (5/35)
T3	150.00 ± 39.32	40.00% (14/35)	42.86 ± 10.51	20.00% (7/35)

**Note:** Data are expressed as mean ± SEM. In the same column of data, the difference in shoulder mark letters means a statistically significant difference (*p* < 0.05), and the absence of shoulder mark letters means that the statistical difference is not significant (*p* > 0.05).

**Table 2:** The serum of the *IL-4* and *IFN-γ* levels in antibody-positive and antibody-negative pigs at different periods.

Age	*IL-4* Level of Serum (ng/mL)		*p*-Value	*IFN-γ* Level of Serum (ng/mL)		*p*-Value
	P	N		P	N	
day 110	181.56 ± 9.62	150.30 ± 15.66	0.103	186.89 ± 32.99	147.74 ± 12.48	0.492
day 185	133.02 ± 11.54	119.82 ± 15.31	0.489	155.16 ± 12.77	142.52 ± 15.45	0.533

**Note:** Data are expressed as mean ± SEM. In the same row of data, the difference in shoulder mark letters means a statistically significant difference (*p* < 0.05), and the absence of shoulder mark letters means that the statistical difference is not significant (*p* > 0.05).

**Table 3:** The serum of the *GH* and *IGF-1* levels in antibody-positive and antibody-negative pigs at different period.

Age	*GH* Level of Serum (ng/mL)		*p*-Value	*IGF-1* Level of Serum (ng/mL)		*p*-Value
	P	N		P	N	
day 110	43.27 ± 2.53	39.93 ± 2.97	0.160	186.83 ± 14.92	166.25 ± 16.99	0.453
day 185	39.41 ± 3.07	35.29 ± 4.24	0.256	157.54 ± 17.17	161.91 ± 14.57	0.857

**Note:** Data are expressed as mean ± SEM. In the same row of data, the difference in shoulder mark letters means a statistically significant difference (*p* < 0.05), and the absence of shoulder mark letters means that the statistical difference is not significant (*p* > 0.05).

**Table 4:** Mean weight and daily gain of each period after immunisation of fattening pigs with different doses of SS DNA vaccine.

Groups	Weight (kg)			Daily Gain (kg/d)		
	Day 50	Day 110	Day 185	Day 50 to 110	Day 110 to 185	Day 50 to 185
T1 (n = 39)	11.18 ± 0.917	58.16 ± 0.667 ^ab^	108.75 ± 0.973 ^a^	0.73 ± 0.012 ^a^	0.73 ± 0.010 ^a^	0.74 ± 0.012 ^a^
T2 (n = 35)	12.09 ± 0.965	58.63 ± 0.696 ^ab^	108.74 ± 1.017 ^a^	0.74 ± 0.012 ^a^	0.72 ± 0.011 ^ab^	0.71 ± 0.012 ^ab^
T3 (n = 35)	11.67 ± 0.961	59.44 ± 0.688 ^a^	110.88 ± 1.002 ^a^	0.74 ± 0.012 ^a^	0.74 ± 0.011 ^a^	0.73 ± 0.012 ^ab^
Ctrl (n = 38)	11.33 ± 0.957	56.42 ± 0.681 ^b^	104.98 ± 0.997 ^b^	0.69 ± 0.02 ^b^	0.69 ± 0.010 ^b^	0.69 ± 0.012 ^b^

**Note:** Different superscripts in the same column indicate a significant difference between various groups (*p* < 0.05). Data are expressed as mean ± SEM.

**Table 5:** The daily gain of antibody-positive and antibody-negative pigs at different period.

Growth Period	P (n = 44)	N (n = 103)	*p*-Value
day 50 to day 110 (kg/d)	0.79 ± 0.012 ^a^	0.70 ± 0.007 ^b^	0.00
day 110 to day 185 (kg/d)	0.77 ± 0.011 ^a^	0.71 ± 0.006 ^b^	0.00
day 50 to day 185 (kg/d)	0.75 ± 0.012 ^a^	0.71 ± 0.007 ^b^	0.01

**Note:** Data are expressed as mean ± SEM. In the same row of data, the difference in shoulder mark letters means a statistically significant difference (*p* < 0.05), and the absence of shoulder mark letters means that the statistical difference is not significant (*p* > 0.05).

**Table 6:** Comparison of meat quality and backfat thickness between antibody-positive pigs and antibody-negative pigs.

Items	P	N	*p*-Value
pH_1_	6.81 ± 0.38	6.76 ± 0.25	0.13
pH_24_	5.76 ± 0.64	5.74 ± 0.78	0.71
Meat colour	3.25 ± 0.50	3.20 ± 0.45	0.88
Marbling	1.50 ± 0.58	1.60 ± 0.55	0.80
Cut resistance (N)	26.48 ± 1.31	25.75 ± 1.13	0.51
Moisture (%)	1.63 ± 0.08	1.61 ± 0.15	0.83
Ash (%)	73.23 ± 0.43	72.88 ± 0.33	0.33
Crude protein (%)	21.31 ± 0.37	21.57 ± 0.23	0.37
Intramuscular fat (%)	1.54 ± 0.30 ^a^	1.60 ± 0.21 ^b^	0.13
Backfat thickness (mm)	14.40 ± 0.82	14.64 ± 1.03	0.20

**Note:** Data are expressed as mean ± SEM. In the same row of data, the difference in shoulder mark letters means a statistically significant difference (*p* < 0.05), and the absence of shoulder mark letters means that the statistical difference is not significant (*p* > 0.05).

**Table 7:** Correlation coefficients of antibody titre with daily weight gain and backfat thickness.

Antibody Titre	Daily Gain			Backfat Thickness
	Day 50 to 110	Day 110 to 185	Day 50 to 185	
day 110	0.664 **	0.042	0.396 *	−0.406 *
day 185	0.207	0.447 *	0.446 *	−0.252

**Note:** * indicates a significant difference (*p* < 0.05), ** indicates a significant difference (*p* < 0.01).

## Data Availability

The datasets used and/or analysed during the current study are available from the corresponding author.

## Supplementary Materials

The following supporting information can be downloaded at: https://www.mdpi.com/article/10.3390/ani12223072/s1, Figure S1: Electrophoresis analysis of GS/2SS PCR products extracted from blood and tissue samples of fattening pigs; Figure S2: Electrophoretogram of PCR amplification products of DNA recombinant fragment GS/2SS in soil samples of somatostatin DNA vaccine release environment; Figure S3: Electrophoretogram of PCR amplification products of DNA recombinant fragment GS/2SS in water and fecal samples of somatostatin DNA vaccine release environment.
