# Supplementary material for: Efficacy and Safety of Nasal Immunisation with Somatostatin DNA Vaccine for Growth Promotion in Fattening Pigs
Source: Animals (Basel). 2022 Nov 8;12(22):3072. doi: 10.3390/ani12223072 (PMC9686601; doi:10.3390/ani12223072)
Supplement: Supplementary file 1 [file animals-12-03072-s001.zip › animals-1901571-supplementary.pdf]

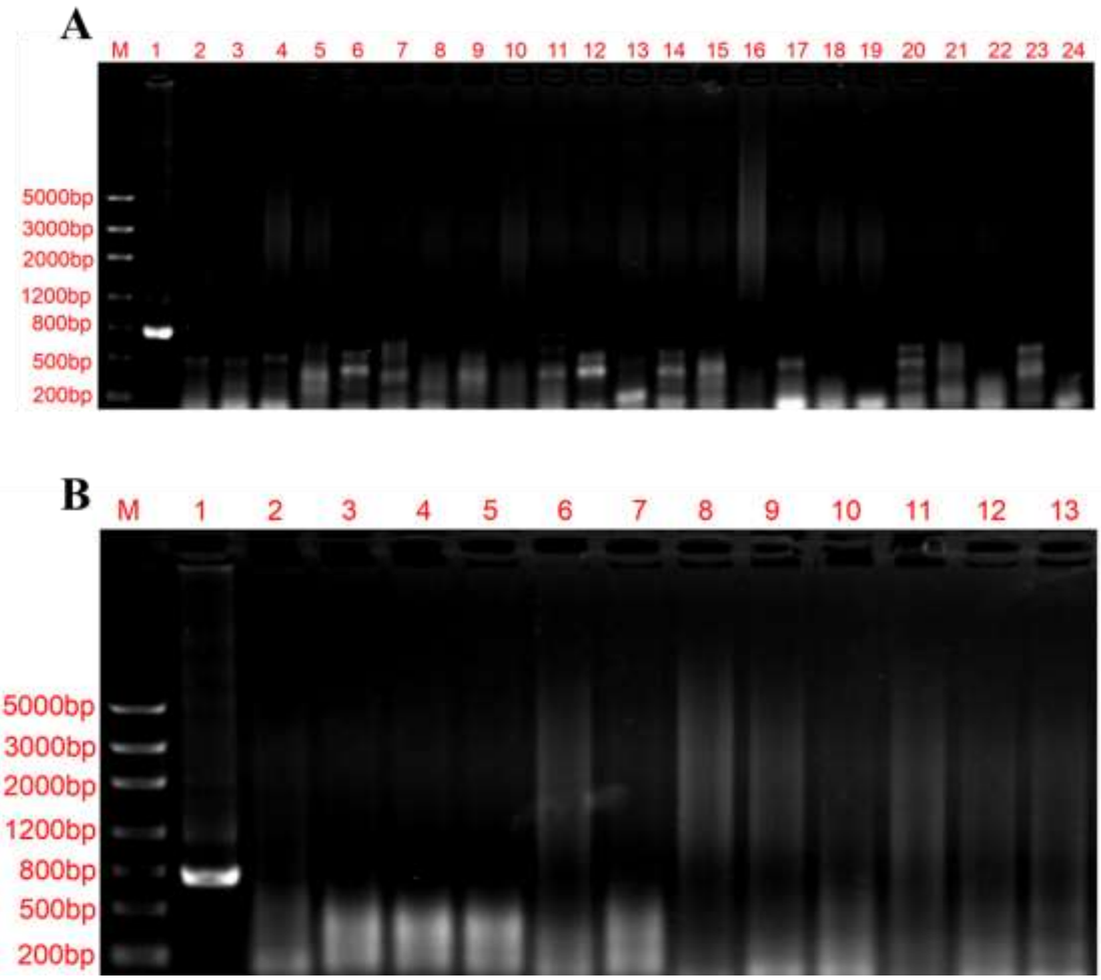

Figure S1. Electrophoresis analysis of GS/2SS PCR products extracted from blood and tissue samples of fattening pigs. A: Electrophoresis of GS/2SS PCR amplification products from whole blood DNA recombinant fragments of test pigs (Lane M: Maeker; Lane 1: positive control; Lane 2-24: Blood sample). B: Electrophoresis of GS/2SS PCR amplification products from antibody-positive pig tissues (Lane M: Marker; Lane 1: Positive control; Lane 2-5: Muscle sample; Lane 6-9: Lung sample; Lane 10-13: Liver sample).

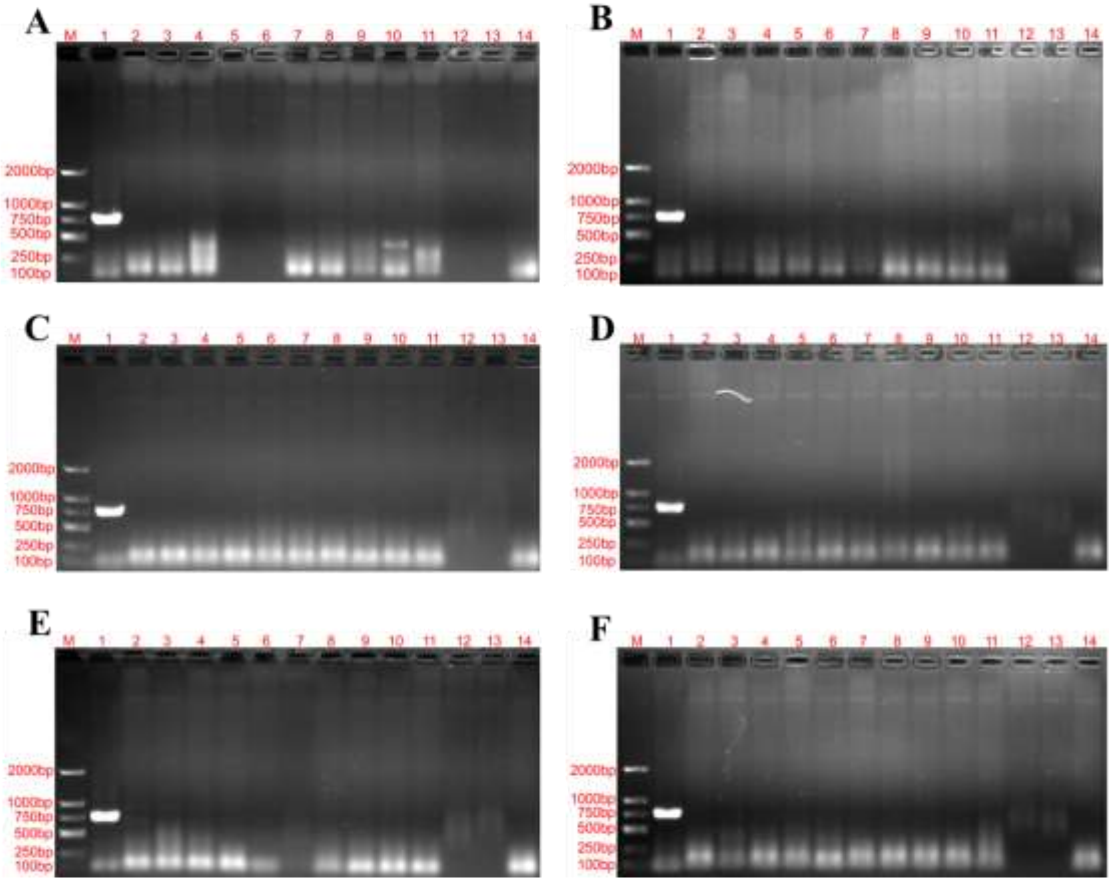

Figure S2. Electrophoretogram of PCR amplification products of DNA recombinant fragment GS/2SS in soil samples of somatostatin DNA vaccine release environment. Figures A, B, C, D, E and F show soil samples from days 3, 5, 7, 10, 15 and 30 after immunisation respectively (Lane M: DNA Marker; Lane 1: Positive control; Lane 2-4: 1m, 3m, 5m east of the barn; Lane 5-7: 1m, 3m, 5m west of the barn; Lane 8-10: 1m, 3m, 5m south of the barn; Lane 11-13: 1m, 3m, 5m north of the barn; Lane 14: 7m at the vent).

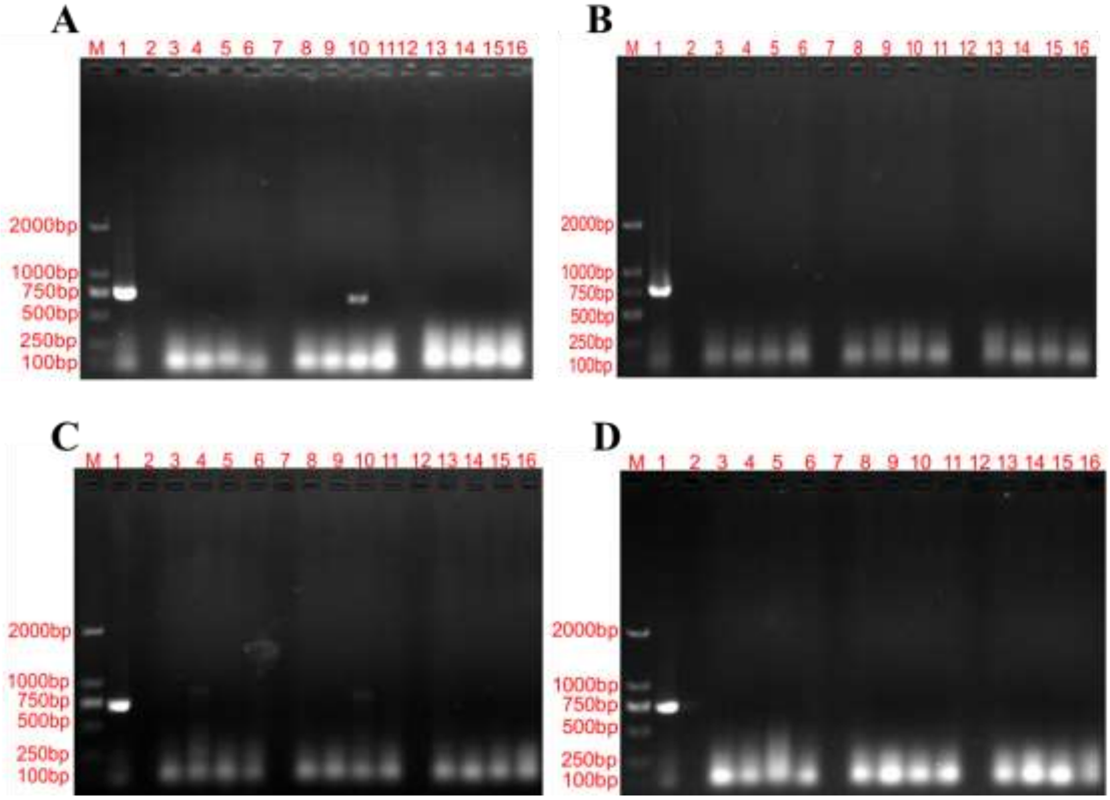

Figure S3. Electrophoretogram of PCR amplification products of DNA recombinant fragment GS/2SS in water and fecal samples of somatostatin DNA vaccine release environment. A (Lane M: DNA Marker; Lane 1: Positive control; Lane 3-6: Water sample on day 3; Lane 8-11: Water sample on day 5; Lane 13-16: Water sample on day 7) and B (Lane M: DNA Marker; Lane 1: Positive control; Lane 3-6: Water sample on day 10; Lane 8-11: Water sample on day 15; Lane 13-16: Water sample on day 30) are water samples. C (Lane M: DNA Marker; Lane 1: Positive control; Lane 3-6: Fecal sample on day 3; Lane 8-11: Fecal sample on day 5; Lane 13-16: Fecal sample on day 7) and D (Lane M: DNA Marker; Lane 1: Positive control; Lane 3-6: Fecal sample on day 10; Lane 8-11: Fecal sample on day 15; Lane 13-16: Fecal sample on day 3) are fecal sample.
